# Metals, Nanoparticles, Particulate Matter, and Cognitive Decline

**DOI:** 10.3389/fneur.2021.794071

**Published:** 2022-01-21

**Authors:** Lilian Calderón-Garcidueñas, Diana A. Chávez-Franco, Samuel C. Luévano-Castro, Edgar Macías-Escobedo, Ariatna Hernández-Castillo, Esperanza Carlos-Hernández, Agustina Franco-Ortíz, Sandra P. Castro-Romero, Mónica Cortés-Flores, Celia Nohemí Crespo-Cortés, Ricardo Torres-Jardón, Elijah W. Stommel, Ravi Philip Rajkumar, Partha S. Mukherjee, Research Universidad del Valle de México UVM Group, Félix Guillermo Márquez Celedonio

**Affiliations:** ^1^Biomedical Sciences, University of Montana, Missoula, MT, United States; ^2^Universidad del Valle de México, Mexico City, Mexico; ^3^Departamento de Psicología, Universidad Autónoma de Coahuila, Saltillo, Mexico; ^4^Universidad Nacional Autónoma de México, Mexico City, Mexico; ^5^Psychology and Medical Consultants, Mexico City, Mexico; ^6^Instituto Politécnico Nacional, Mexico City, Mexico; ^7^Department of Neurology, Dartmouth-Hitchcock Medical Center, Geisel School of Medicine, Lebanon, NH, United States; ^8^Department of Psychiatry, Jawaharlal Institute of Postgraduate Medical Education and Research, Pondicherry, India; ^9^Interdisciplinary Statistical Research Unit, Indian Statistical Institute, Kolkata, India

**Keywords:** Alzheimer's, cognition, metals, nanoparticles, dementia, MoCA, mild cognitive impairment (MCI), PM_2.5_ air pollution

## Abstract

Exposure to metals is ubiquitous and emission sources include gasoline, diesel, smoke from wildfires, contaminated soil, water and food, medical implants, waste recycling facilities, subway exposures, and occupational environments. PM_2.5_ exposure is associated with impaired cognitive performance, neurobehavioral alterations, incidence of dementia, and Alzheimer's disease (AD) risk. Heavy-duty diesel vehicles are major emitters of metal-rich PM_2.5_ and nanoparticles in Metropolitan Mexico City (MMC). Cognitive impairment was investigated in 336 clinically healthy, middle-class, Mexican volunteers, age 29.2 ± 13.3 years with 13.7 ± 2.4 years of education using the Montreal Cognitive Assessment (MoCA). MoCA scores varied with age and residency in three Mexican cities with cognition deficits impacting ~74% of the young middle-class population (MoCA ≤ 25). MMC residents ≥31 years ($\bar{\text{x}}$46.2 ± 11.8 y) had MoCA $\bar{\text{x}}$20.4 ± 3.4 vs. low pollution controls 25.2 ± 2.4 (*p* < 0.0001). Formal education years positively impacted MoCA total scores across all participants (*p* < 0.0001). Residency in PM_2.5_ polluted cities impacts multi-domain cognitive performance. Identifying and making every effort to lower key pollutants impacting neural risk trajectories and monitoring cognitive longitudinal performance are urgent. PM_2.5_ emission control should be prioritized, metal emissions targeted, and neuroprevention interventions implemented early.

## Introduction

Brain metal homeostasis is critical at all stages of development (1–4). Intoxication with heavy metals is a global health problem (5) as is the extensive environmental Al pollution (2) and the metal exposures associated with outdoor and indoor particulate matter (PM) in urban environments (6, 7). Exposure to metals is ubiquitous and emission sources are heterogeneous: gasoline, diesel, alternative mixed biofuels, contaminated soil, water and food, medical implants, waste recycling facilities, subway, lubricating oils, and occupational environments (8–12).

Emissions from internal combustion engines burning fossil fuels in motor vehicles and equipment account for a large fraction of total regional and urban pollution (13). Exposures to air pollutants, including fine particulate matter (PM_2.5_) and their metal content (7, 9, 11) negatively impact cognitive abilities across ages, in the short and long term (14–17). Residents in Metropolitan Mexico City (MMC) are chronically exposed to complex emission mixtures of fine particulate matter (PM_2.5_) containing toxic combustion and industrial metals (18). In an olfactory bulb and frontal cortex autopsy study of 12 low air pollutant controls vs. 47 MMC children and young adults of age 33.06 ± 4.8 years, inductively coupled plasma mass spectrometry and real-time PCR to evaluate COX2, IL1β, and DNA repair genes, highly exposed residents had higher concentrations of manganese (*p* = 0.003), nickel and chromium (*p* = 0.02) along with higher frontal COX2 mRNA (*p* = 0.008) and IL1β (*p* = 0.0002), and olfactory bulb COX2 (*p* = 0.005) indicating neuroinflammation (18). Frontal metals correlated with olfactory bulb DNA repair genes and with frontal and hippocampal inflammatory genes (18). We have recently described in 202/203 consecutive forensic autopsies of MMC 25.3 ± 9.2 years old, including 44 children, age 12.9 ± 4.9 years, the presence of neuropathological markers of Alzheimer's disease (AD): hyperphosphorylated tau (P-tau) and amyloid-β (Aβ) starting in 11-month-old babies (19), along with frontal cortex upregulation of gene clusters IL1, NFκB, TNF, IFN, and TLRs and downregulation of the prion-related protein [PrP(C)] (20). High redox, combustion, and friction-derived magnetite nanoparticles (NPs) and metals, such as Ti and Al, were documented in all MMC brains and were associated with an overlap of aberrant proteins (P-tau, Aß, α synuclein, TDP-43) (21). In a 517 cohort of young individuals, age 21.60 ± 5.88 years, with lifetime exposures to PM_2.5_ (22), we described cognitive impairment in 55% of the population screened with the Montreal Cognitive Assessment (MoCA).

The epidemiological literature supports an association between dementia, traffic, air pollution, and the risk of dementia, particularly AD (14–17). Jung and co-workers (14) described 138% increased risk of AD per 4.34 μg/m^3^ above the U. S. EPA PM_2.5_ annual standard (12.0 μg/m^3^ annual mean averaged over 3 years)and living closer to high traffic roads is associated with increased AD risk (15). Urban populations are exposed to neurotoxicants, including metals, through complex mixtures of air pollutants, diverse regional emission sources, and occupational exposures (23–25). MMC residents have high concentrations of metals vs. low pollution controls in areas such as frontal and olfactory bulbs and millions of combustion-friction derived, metal-rich nanoparticles in critical brain areas (18, 21, 25).

We have one primary aim for this study: To document in 336 Mexican young urbanites their performance in MoCA across three cities with complex patterns and sources of air pollutants, including PM_2.5_ above and below the current U.S. EPA standards (26). We strongly argue and emphasize that in a young population with 13.7 ± 2.4 years of formal education, any scoring ≤ 25 obligates further testing beyond MoCA. Early identification of cognitive impairment in young air pollution–exposed urbanites and understanding the relationship between tau and amyloid pathology (19–21), cognitive impairment (22), and the work in progress criteria to define AD, including the NIA-AA research framework “*defining AD by its underlying pathological processes that can be documented by post-mortem examination or in vivo by biomarkers,”* the ATX (N) system where X includes potential novel biomarkers for pathophysiological mechanisms, and the recent recommendations of the International Working Group on how biomarkers should and should not be used for diagnosing AD in a clinical setting, are all at the core of our research efforts (27–29).

## Methods

### Air Quality Data

We studied three urban areas including MMC, the Port of Veracruz in the coastal central part of the Gulf of Mexico, and a control city with PM_2.5_ ( ≤ 2.5 μm diameter particles) below the current U.S. standards: Hermosillo in the northern border state of Sonora with the United States. We focused on PM_2.5_ and worked with both the respective 24-h and annual averages for 2019 using all the available data for the study. To evaluate the state of the air quality during the study period, we used PM_2.5_ U.S. EPA [National Ambient Air Quality Standard (NAAQS)] and the WHO guidelines, given that Mexican standards are less stringent as shown in Supplementary Table 1.

Metropolitan Mexico City has a population of ~22 million people, it lies on a semi-closed basin surrounded by mountains. Mobile sources strongly contribute to the burden of emitted pollutants. More than 6 million gasoline-powered cars with a mean age of ~6.5 years circulate every day in MMC, while 350,000 much older diesel heavy-duty vehicles (HDVs) (~13.5 years) are also in circulation (30). The majority of HDVs in circulation have obsolete technologies or lack technologies to control emissions. As a result, HDVs in MMC are the main emitters of nitrogen oxides (NOx), PM_2.5_, and black carbon (30). MMC has an air quality monitoring network of more than 30 stations with PM_2.5_ systematic measurements starting in 2004, with current trends suggesting increments (31). Key for the health effects, is the fact that ~75% of PM_2.5_ is smaller than 1 micron (PM_1_), which includes the ultrafine particles ( ≤ 100 nm) (23). Over 47% of the MMC, PM_2.5_ comprises organic aerosols (OA), 30% of secondary organic aerosols (SOA), 12% black carbon (BC), 7% soil components, 1% metals, and the rest being mineral components (32). OA includes primary organic aerosols (POA) i.e., primary hydrocarbon-like compounds, polycyclic aromatic hydrocarbons (PAH), and their nitrogen derivatives (32). PAH, the semi-volatile complex organic compounds associated with incomplete combustion processes of fossil fuels and biomass, have been increasing ~150% in Mexico City in the last 5 years (33). Critical to this study, anthropogenic metals, i.e., chromium, zinc, copper, lead, vanadium, antimony, and barium, are present in the PM_2.5_ mass (34). Metals are largely associated with industrial and mobile sources (34). Elements associated with road traffic include Cr, Mn, Zn, and Pb associated with engine emissions, abrasion of tires and brake pads, while subway exposures are also high in PM_2.5_ ranging from 60 to 93 μg/m^3^ and high Fe, Cu, Ni, Cr, and Mn concentrations (11, 30, 31). V and Ni are tracers of long-range transport from the use of heavy fuel oil in the northern industrial area of Tula in the State of Hidalgo. MMC residents are exposed to complex mixtures of air pollutants, including metals, representing a serious health problem for everyone regardless of age, gender, and socioeconomic status (23, 24, 30–34).

The Port of Veracruz has an estimated population of ~850,000 people. The port has flatland topography and is hot and humid; most of the time receives winds from the sea that supports continuous urban ventilation. The port activities (ships, cargo trucks, cranes, etc.) add to those of the urban activity resulting in PM_2.5_ and SO_2_ emissions (35–37).

Hermosillo is a city in the southern extreme of the Sonora Desert in northwestern Mexico at 200 m above sea level, with good ventilation all year long. It has a population of ~813,000 people and air pollution sources are mostly traffic, industry and agricultural activities, and unpaved roads being a strong dust source (38). The measurement of air quality in Hermosillo began in 1989 with three stations sampling total suspended particles (TSP), which later were complemented with PM_10_ and gaseous air pollutants. By 2016, continuous air quality monitoring included automatic measurement of PM_10_ and PM_2.5_ (39). Re-suspension of dust in unpaved streets is the main air pollution source leading to sustained high levels of PM_10_ (38). Fine particles in Hermosillo are generated mainly by traffic emissions and some industries and they comprise < ~20% of the PM_10_. We documented the PM_2.5_ population exposure during the year 2019 from continuous hourly averaged PM_2.5_ data for MMC and Hermosillo (30, 38). However, since we have no PM_2.5_ monitoring stations in Veracruz, we estimated the respective seasonal and annual average concentrations from spatial satellite-derived distribution maps for the Mexico region as per Van Donkelaar et al. (40). These authors combined geosciences–statistical methods with satellite information, air quality data, and other models to obtain global and regional medium- and long-term average estimates of PM_2.5_ (40). To characterize the PM _2.5_ air quality data in MMC, Hermosillo, and Veracruz, we processed the PM_2.5_ monitoring data for MMC and Hermosillo, and the information from satellite-derived distribution maps of surface PM _2.5_ for the study year and several Veracruz annual trend periods.

### Study Population and Demographics

The research was done in accordance with the ethical standards of the Revised Helsinki Declaration of 2000, this study was approved by ethical and research committees of the University of Montana (IRB#206-R-09 and IRB#185-20) and Universidad del Valle de Mexico (March 16, 2016) and a written informed consent obtained from all participants. Volunteers were identified by word-of-mouth advertising from previous participants in our clinical studies through social media, churches, and work sites. This is a cross-sectional study of 336 clinically healthy, middle-class Mexican participants who fulfill the inclusion criteria [negative family history of AD or Parkinson's disease (PD), no history of hospitalizations, chronic degenerative diseases, head trauma, and no prescribed or over-the-counter medications for the year previous to this study]. Subjects completed a medical examination including a complete clinical history and a physical examination and were considered clinically healthy. None of the participants were smokers or past smokers, had an alcohol use disorder, as per DSM-5, or consumed illicit drugs.

### MoCA Administration and Scoring

The Spanish version of MoCA was used in this study and applied by a professor certified by MoCA (MXCRECE191274-01). We selected to apply normal cognition scores of 26–30, 24–25 as scores for mild cognitive impairment (MCI) and ≤ 23 for serious cognitive impairment, based on previous literature and the DSM-5 (41–44). MoCA assesses global cognitive function and contains 10 subtests (41). MoCA scores were converted into six index scores based on the combinations used by Julayanont et al. and Petersen (42, 45). The Executive Index Score (EIS) was the sum of trail making, clock drawing, digit span forward and backward, letter A tapping, serial 7's subtraction, word fluency and abstraction (total score 13); Language Index Scores (LIS): animal naming, sentence repetition, and word fluency (total score 6); Visuospatial Index Score (VIS): cube copy, clock drawing, and animal naming (total score 7); and Attention Index Score (AIS): digit span forward and backward, letter A tapping, serial 7's subtraction, sentence repetition, the 10 words recalled at both immediate recall trials (total points 18). Petersen's original Delayed Recall Score plus VIS, EIS, and LIS were also used (45).

### Statistical Analysis

We first calculated the summary statistics of the MoCA scores in the individuals grouped by residency: MMC, Hermosillo, and Veracruz age ≤ 30 years and MMC, Hermosillo, and Veracruz age ≥ 31 years. Then, for the initial investigation, we tested the equality of mean scores of each meaningful pair using the two-sample *t*-tests. We performed a similar procedure for index scores. We also calculated adjusted *p*-values after removing the linear effects of age, gender, BMI, and education years. Next, we created three groups: normal, MCI, and serious cognitive impairment: the cutoff points based on total MoCA scores, normal ≥ 26, MCI 24–25 and ≤ 23. We tested the equality of mean scores of each meaningful pair. We calculated the index scores in each group as well. We also fitted multiple linear regression of the total MoCA score on age, BMI, gender, and education years. We run the regression models in each of the aforementioned sections of our data. For cognitive analyses, we used robust locally weighted smoothing scatter plots. We performed the statistical analyses using Excel and the statistical software “R” (http://www.r-project.org/).

## Results

### Air Pollution Data

**Figure 1:**
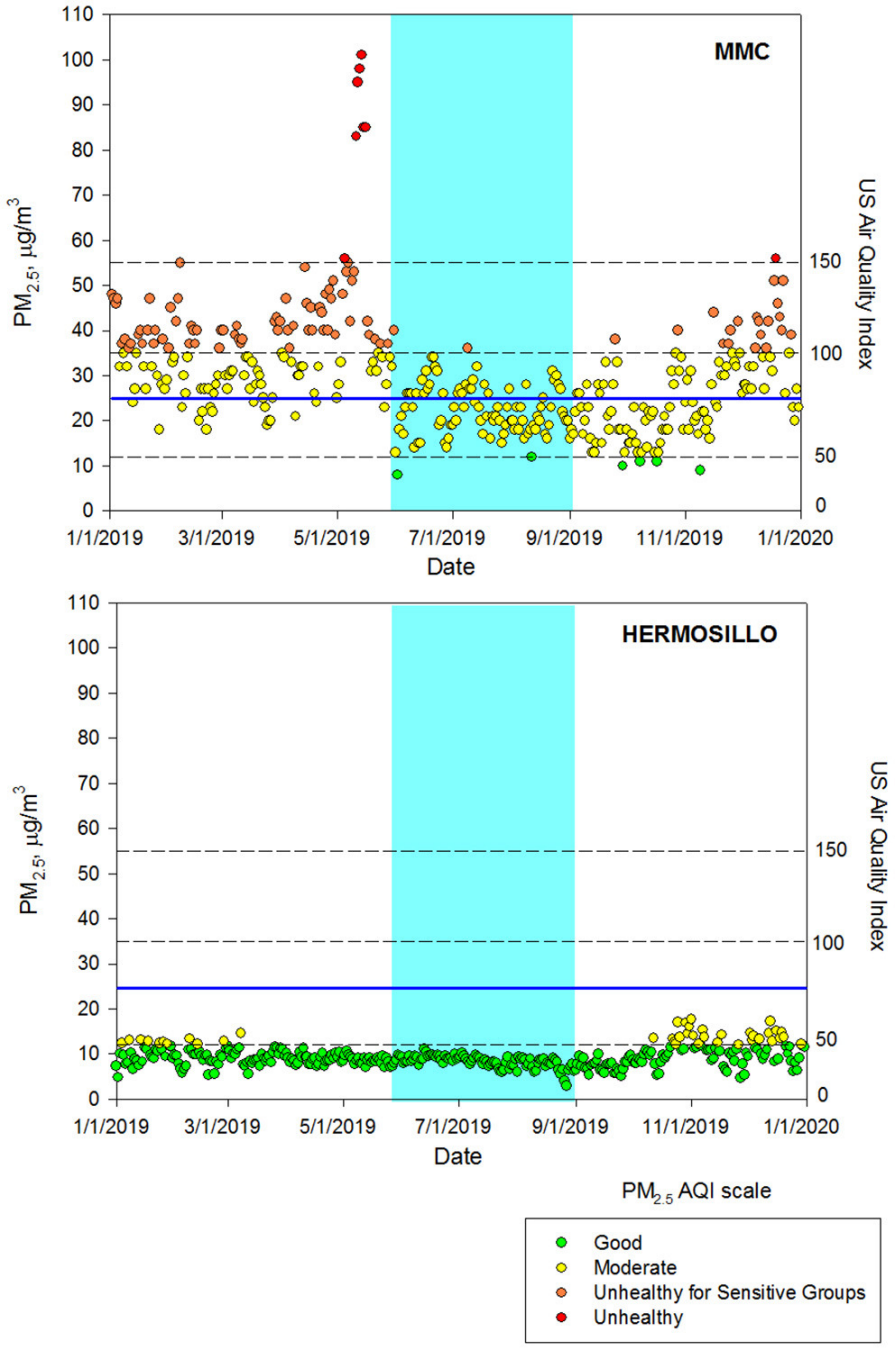
Time-series of PM_2.5_ 24-h averages at MMC and Hermosillo from January 1, 2019 to May 31, 2020, classified according to the U.S. EPA AQI index*. *The blue continuous line depicts the 24-h average guideline of the WHO. The blue shade area represents the period during the MoCA evaluations. Air quality data are available from Sistema de Monitoreo Atmosferico del Gobierno de la Ciudad de México (http://www.aire.cdmx.gob.mx/default.php) and Red Universitaria de Observatorios Atmosféricos de la Universidad Nacional Autónoma de México (https://www.ruoa.unam.mx/).

Figure 1 shows the time-series for the daily maximum of PM_2.5_ 24-h averages for MMC and Hermosillo classified according to the U.S. EPA AQI-Index for 2019 and up to May 2020.

A summary of the daily averages during the 2019 MoCA evaluation period and the three trends of three quinquennial periods between 2000 and 2016 for MMC, Hermosillo, and the respective estimates for Veracruz are shown in Table 1. A comparison of the annual averages obtained from measurements with those in the maps of reference (40) showed a reasonable agreement among them.

**Table 1:** Summary of the 24-h PM_2.5_ averages in μg/m^3^ for the 2019 period of MoCA evaluations and the annual periods: 2000-2004; 2006-2010, and 2012-2016 for MMC, the City of Hermosillo and the Port of Veracruz.

**Urban area**	**MoCA evaluation summer 2019**	**2019**	**Five-year period**		
			**2000–2004**	**2006–2010**	**2012–2016**
MMC	22 μg/m^3^	30 μg/m^3^	36 μg/m^3^	26 μg/m^3^	27 μg/m^3^
Veracruz	13 μg/m^3^	16 μg/m^3^	13 μg/m^3^	12 μg/m^3^	10 μg/m^3^
Hermosillo	8 μg/m^3^	9 μg/m^3^	11 μg/m^3^	9 μg/m^3^	9 μg/m^3^

As shown in Figure 1, most of the MMC daily PM_2.5_ averages during 2019 were above the respective WHO guideline and 25% of the days were above the U.S. EPA NAAQS, which equals the 100-AQI index limit (Moderate to Unhealthy Air Quality). MMC and Veracruz exceeded the PM_2.5_ U.S. EPA NAAQS annual average and the guideline of the WHO of 12 and 10 μg/m^3^, respectively (Table 1; Supplementary Table 1). In contrast, in the control city Hermosillo, all days in 2019 were below both, the WHO guideline and the U.S. EPA NAAQS. The PM_2.5_ 24-h average levels in MMC were mostly in the range 50-100 of the AQI index (Moderate) while in Hermosillo practically all days had good air quality. The estimated daily averages for the Port of Veracruz for the same period ranged around the 50-AQI, which is the boundary between Good and Moderate air quality.

### Demographic and MoCA Results

The 336 participants had an average age of 29.2 ± 13.3 years, with 13.7 ± 2.4 years of formal education and a BMI of 25.3 ± 3.9 kg/m^2^ (Table 2). The mean MoCA score for the entire group was 23.3 ± 3.2. MoCA total scores and Cognition Index scores in each city in ≤ 30 and ≥ 31-year-olds are seen in Table 2. The MMC MoCA score for 83 subjects ≥31 years (average age 46.4 ± 11.8 years) was 20.4 ± 3.4 (Table 2). In sharp contrast, significantly higher MoCA scores were seen in adults ≥ 31 years living in Hermosillo vs. MMC: 25.2 ± 2.4 vs. 20.4 ± 3.4, *p* < 0.0001. Eighty-four percent of MMC residents ≥31 years had MoCA scores ≤ 23.

**Table 2:** Summary of MoCA scores, age, BMI, education years, and cognitive domain scores.

**Residency**	**MoCA scores**	**Average age years**	**BMI**	**Education years**	**Memory**	**EIS**	**LIS**	**VIS**	**AIS**	**OIS**	**Delay recall + EIS + VIS + LIS**
						**Total:13**	**Total:6**	**Total:7**	**Total:18**	**Total:6**	**Total:31**
						**CUTOFF**	**CUTOFF**	**CUTOFF**	**CUTOFF**	**CUTOFF**	**CUTOFF**
						**SCORE**	**SCORE**	**SCORE**	**SCORE**	**SCORE**	**SCORE**
						**10.5**	**5.5**	**5.5**	**16**	**5.5**	**24**
MMC ≤ 30 y *N*:150	24.2 ± 2.6	21.5 ± 3.5	24.2 ± 3.2	13.6 ± 1.7	2.7 ± 1.4	10.7 ± 1.6	5.1 ± 0.8	5.8 ± 1.1	16.4 ± 1.2	5.8 ± 0.4	24.4 ± 3.2
Veracruz ≤ 30 y *N*:48	24.3 ± 2.7	20.1 ± 1.8	24.5 ± 3.3	13.0 ± 0.2	4.5 ± 0.7	10.4 ± 1.9	5.8 ± 0.4	5.9 ± 0.9	16.8 ± 1.2	5.7 ± 0.4	26.8 ± 2.6
Adjusted *p*-value MMC vs. Veracruz	0.5	NA	NA	NA	<0.0001	0.6	<0.0001	0.3	0.01	0.8	<0.0001
Hermosillo ≤ 30 y *N*:22	24.7 ± 2.1	19.3 ± 1.3	21.9 ± 2.7	13.7 ± 0.6	3.1 ± 1.3	11.1 ± 1.1	5.2 ± 0.5	5.3 ± 0.9	16.5 ± 1.2	5.9 ± 0.2	24.9 ± 2.4
Adjusted *p*-value MMC vs. HER	0.4	NA	NA	NA	0.07	0.4	0.4	0.03	0.6	0.1	0.4
MMC ≥ 31 y *N*:83	20.4 ± 3.4	46.4 ± 11.8	27.8 ± 3.9	13.2 ± 3.3	1.4 ± 1.4	9.2 ± 2.2	4.3 ± 1.5	4.4 ± 1.7	15.3 ± 1.7	5.7 ± 0.4	19.5 ± 4.8
Veracruz ≥ 31 y *N*:19	24.0 ± 2.7	38.1 ± 7.2	26.9 ± 2.6	17.1 ± 3.2	2.8 ± 1.1	10.0 ± 1.4	4.6 ± 1.1	5.6 ± 1.1	15.7 ± 1.5	5.8 ± 0.3	23.3 ± 3.2
Adjusted *p*-value MMC vs. VER	0.2	NA	NA	NA	0.04	0.2	0.2	0.2	0.3	0.7	0.9
Hermosillo ≥ 31 y *N*:13	25.2 ± 2.3	44.0 ± 7.2	26.9 ± 4.3	15.2 ± 2.8	3.3 ± 1.7	10.9 ± 1.3	5.7 ± 0.4	5.0 ± 1.1	17.3 ± 1.0	5.9 ± 0.2	25.0 ± 3.1
Adjusted *p*-value MMC vs. HER	<0.0001	NA	NA	NA	0.0004	0.08	0.0078	0.4	0.001	0.2	0.0005

*Executive Index Score (EIS) is the sum of Trail making, clock drawing, digit span forward and backward, letter A tapping, serial 7's subtraction, word fluency, and abstraction. Language Index Scores (LIS): animal naming, sentence repetition, and word fluency. Visuospatial Index Score (VIS): cube copy, clock drawing, and animal naming. Attention Index Score (AIS): digit span forward and backward, letter A tapping, serial 7s subtraction, sentence repetition, and Words Recalled in Both Immediate Recall Trials. The Orientation Index Score (OIS) includes all the Orientation items (0–6 points). Summary scores: delay recall+ EIS, VIS, and LIS*.

Analysis of the Cognition Index Scores in the groups with normal cognition NC ≥26, MCI 24–25 and ≤ 23 showed the progressive changes with MCI subjects showing significant deficits in the Language Index Scores (LIS) (animal naming, sentence repetition, and word fluency), while subjects with ≤ 23 score had all Cognition Index Scores compromised except for Orientation (Table 3).

**Table 3:** Cognitive domains index scores.

**Groups based on MoCA total scores Mean ± SD**	**EIS**	**LIS**	**VIS**	**AIS**	**Orientation**	**Delay recall+EIS+VIS+LIS (45)**
	**Total:13**	**Total:6**	**Total:7**	**Total:18**	**Total:6**	**Total:31**
	**CUTOFF**	**CUTOFF**	**CUTOFF**	**CUTOFF**	**CUTOFF**	**CUTOFF**
	**SCORE**	**SCORE**	**SCORE**	**SCORE**	**SCORE**	**SCORE**
	**10.5**	**5.5**	**5.5**	**16**	**5.5**	**24**
MoCA ≥26 Normal cognition (27.1 ± 1.1) Age 25.3 ± 9.3 y *n*:88 (27M, 61F)	11.9 ± 0.9	5.6 ± 0.5	6.2 ± 0.8	17.3 ± 0.7	5.9 ± 0.2	27.7 ± 1.6
MoCA 24–25 MCI (24.4 ± 0.5) Age 24.4 ± 7.8 y *n*:93 (30M, 63F)	10.8 ± 1.1	5.2 ± 0.7	5.6 ± 0.9	16.6 ± 1.0	5.8 ± 0.3	25.0 ± 1.6
MoCA ≤ 23 (20.5 ± 2.4) Age 34.4 ± 15.8 y *n*:155 (32M, 12F)	9.1 ± 1.9	4.5 ± 1.3	4.8 ± 1.5	15.3 ± 1.4	5.7 ± 0.5	20.3 ± 4.0

**Figure 2:**
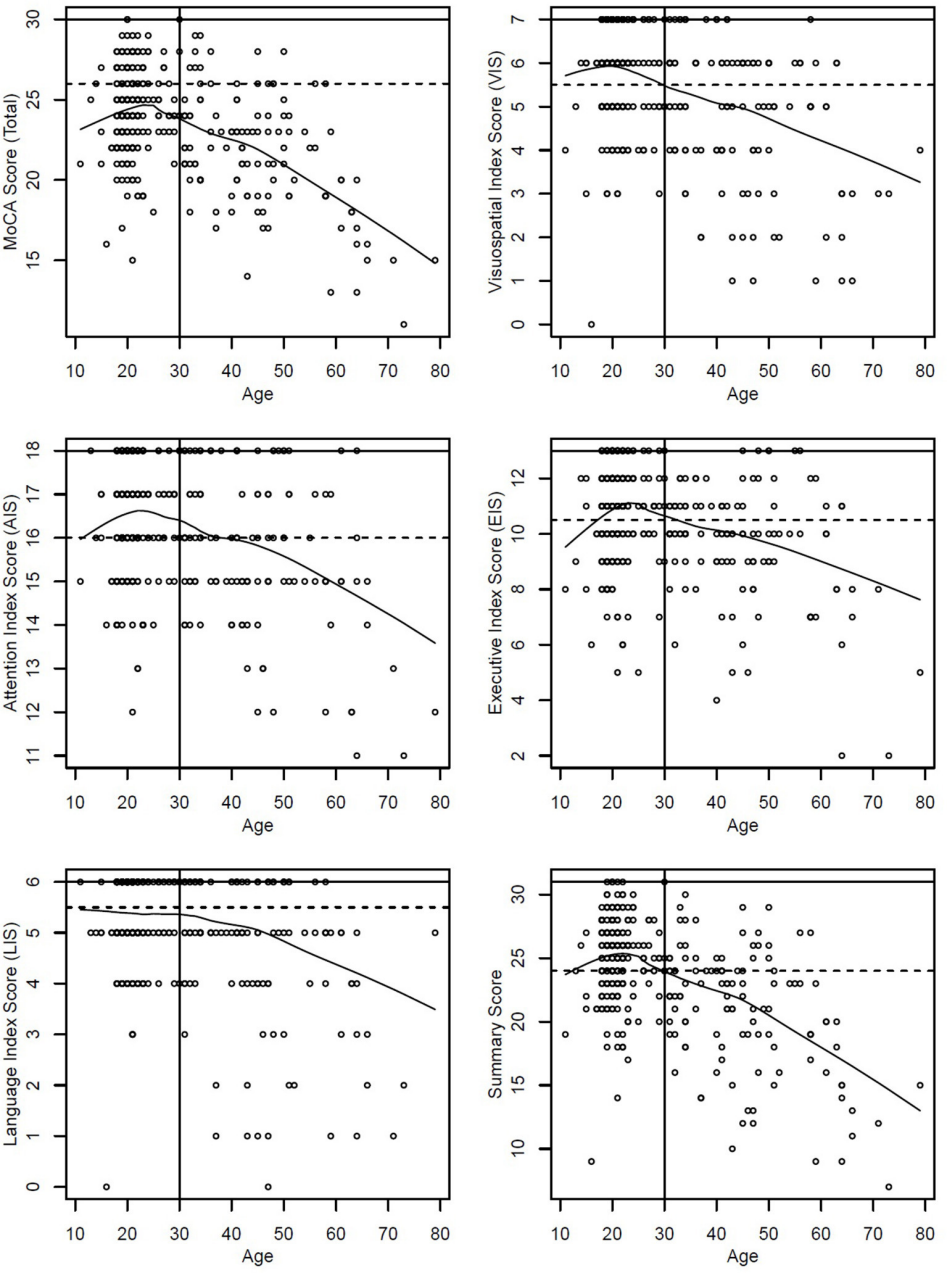
The cognitive decline for the entire group is shown for MoCA total score, and for each Cognitive Index Scores (solid line maximum score and dashed line cutoff score).

The number of formal education years impacted positively the MoCA total scores across all participants (*p* < 0.0001), while age impacted negatively across the entire group (*p* < 0.0001). Gender and BMI were not significant (*p* = 0.94 and *p* = 0.83). Figure 2 shows the cognitive decline for the entire group in the MoCA total score and the Cognition Index Scores.

## Discussion

In this study of clinically healthy middle-class individuals with lifelong exposures to complex mixtures of air pollutants, including PM_2.5_ with high metal concentrations, cognition deficits were documented in 74% of the population. MoCA scores ≤ 23 were documented on subjects with an average age of 34.4 ± 15.8 years.

The problem is deeply concerning for highly exposed MMC residents 31 years and older: 84% with an average age of 46.6 ± 11.8 years had average scores of 20.4 ± 3.4. The National Institute of Statistics and Geography (46) released the March 2–27, 2020, Census data, and Mexico City (part of the Metropolitan Area) with 9,209,944 residents has the oldest average population in the country: 35 years and an average of 9 years of formal education, which severely heightens the cognitive problem as the individuals in this study had 13.7 ± 2.4 years of formal education.

In this work, we are documenting a striking progression of specific domain deficits from the third decade of life, onward. The progression to scores ≤ 23 is characterized by all the Cognitive Domain Index scores below the cutoff scores except Orientation. The targeted domains involved, point toward progressive brainstem involvement and the temporal–parietal–frontal circuit involvement (21, 47–52). The targeted domains information is critical, because we have shown in consecutive forensic autopsies of MMC individuals, extensive brainstem pathology including progressive quadruple abnormal proteins, and neuroinflammation, and clinically: abnormal brainstem auditory-evoked potentials, and stress and sleep behavior disorders (21, 53, 54). We suggest that for MMC residents, cognitive deficits at a young, productive age could be associated with the progression from tau pre-tangles to neurofibrillary tangles (NFT) stages I–II to NFT stages III–V in the first four decades of life, as reported in forensic autopsies (19). It is important to remember that in 99.5% of the MMC forensic unselected autopsies, we were able to stage for P-tau and the Aβ phases (19, 21, 55–59). Thus, regarding the question of Del Tredeci and Braak (60): *To stage, or not to stage*, we strongly support that staging at early ages using forensic samples is critical in heavily polluted cities to define the percentage of cases with aberrant protein neuropathology hallmarks in each population and specifically starting in pediatric ages (19, 21). Staging is a very helpful guide precisely regarding the topographic extent of the abnormal proteins and the expected cognitive/neurological/and brain MRI alterations across the first four decades of life. Careful staging and selection of sampled regions, measuring the concentrations of magnetic nanoparticles, and defining their metal and non-metal content in fresh samples along with immunohistochemistry have confirmed the overlap between AD, PD, and TDP-43 pathology in MMC young urbanites (21, 61). We agreed with Jack (62) regarding the validity of the preclinical AD concept; hence, it is very important to include early cases associated with pollution exposures in the research frame. The concept of AD as defined by the National Institute on Aging and Alzheimer's Association Research Framework (27) has given rise to significant debate and discussion of the use of biomarkers in the clinical setting (28, 29) and the length of the prodromal stage in heavily pollution-exposed subjects (19–22, 63, 64).

This work has five key information pieces: i. The cognition deficits impact ~74% of the young middle-class population. ii. MMC residents older than 31 years (average 46.2 ± 11.8 years), are already showing strikingly low MoCA scores on average 20.4 ± 3.4, iii. Formal education years positively impact MoCA total scores across all participants (*p* < 0.0001), iv. Getting older in a sustained PM_2.5_ environment above the current U.S. EPA annual standard worsens cognition deficits, and v. Gender and BMI do not impact MoCA scores.

There is no doubt millions of Mexicans and people across the world are exposed to complex mixtures of air pollutants and the interplay of air pollution, diverse sources of neurotoxicants, lifestyle, socioeconomic factors, chronic social stress, violence, etc., likely signal the trajectory of young people toward progressively worse cognitive impairment (65–70).

### Advantages and Shortcomings of This Study Relative to Other Studies

A major advantage of our research design is the access to subjects with the same socioeconomic status and ethnicity, enabling us to rule out the possibility that these key variables will modify our results across different urban areas. We have a detailed description of forensic autopsies in young individuals with quadruple brain aberrant pathology and no extra-neural pathology, that allowed us to put forward potential associations with MoCA in MMC residents and indirectly with residents across the country (19, 21).

The study has shortcomings. Our major gap is that we based our exposures to air pollution on PM_2.5_ environmental outdoor data and included only non-smokers; however, we did not monitor for other sources of PM_2.5_, including cooking, traveling patterns, proximity to emission sources, etc.

## Conclusions

Urban populations across the world are exposed from early uterine life on, to complex mixtures of neurotoxicants. MMC residents are an example of sustained exposures to PM_2.5_ containing toxic metals and highly reactive metal nanoparticles capable of reaching the brain. The application of a brief cognitive instrument (MoCA) allows trained health providers to identify individuals with normal cognition and obligates us to go further into their cognitive neuro-evaluation when we encounter scores ≤ 25 in the setting of healthy individuals with more than 12 years of formal education.

Our results are disturbing, in the entire group only 26% had normal cognition, so the issue should be an urgent public health research priority and the impact on health, educational, social, economy, and the judicial systems ought to be a serious concern. Identifying and making every effort to lower key pollutants impacting neural risk trajectories and monitoring cognitive longitudinal performance, would greatly facilitate multidisciplinary interventions for early neurodegenerative diseases in high-risk young world populations. We strongly support that air pollution, metals, and nanoparticles, play key roles in the development of diseases, such as Alzheimer, thus keeping PM_2.5_ exposures below current standards is crucial.

## Data Availability Statement

The original contributions presented in the study are included in the article/Supplementary Material, further inquiries can be directed to the corresponding author/s.

## Ethics Statement

This research was done in according with the ethical standards of the Revised Helsinki Declaration of 2000, also approved by ethical and research committees of the University of Montana (IRB#206-R-09 and IRB#185-20), and Universidad del Valle de Mexico (March 16, 2016). All participants provided their written informed consent to participate in this study

## Author Contributions

LC-G study concept and design, analysis and interpretation of data, writing, drafting, revising the manuscript, study supervision, coordination, and funding. RR and ES analysis and interpretation of data, writing, drafting, and revising the manuscript. PM did the statistical analysis, writing, drafting, and revising the manuscript. RT-J wrote the air pollution sections. All author's including the Research Universidad del Valle de México UVM Group acquisition of data, study supervision, coordination, analysis, and interpretation of data.

## Funding

This work was supported by the SEP-CONACYT project 255956 G7 CB-2015-01.

## Conflict of Interest

The authors declare that the research was conducted in the absence of any commercial or financial relationships that could be construed as a potential conflict of interest.

## Publisher's Note

All claims expressed in this article are solely those of the authors and do not necessarily represent those of their affiliated organizations, or those of the publisher, the editors and the reviewers. Any product that may be evaluated in this article, or claim that may be made by its manufacturer, is not guaranteed or endorsed by the publisher.

## Research Universidad del Valle de México UVM Group

Félix Guillermo Márquez Celedonio, Nora B. Vacaseydel-Aceves, Sandra Carrillo-Ibarra, Jorge Roura-Velasco, Joaquín Vázquez-Cruz, Lucero Aída Juárez-Herrera- Y-Cairo, Noelia Guadalupe Fierro-Fimbres, Karina Águila-Castellanos, Abel Arballo-Romero, Nilza Burruel-DeLaCruz, Kristel Castelar-Ibarra, Beatriz Cuéllar-Figueroa, Priscilla Moreno-Barceló, José Luis Romero-Romero, Jaquelinne Sedano-Benítez, Viviana Moreno-Monreal, Fernanda Dávila-Ortiz, Silvia Ramírez-Sánchez, Edgar García-Rojas, Rafael Brito-Aguilar, Luis E. Jiménez-Hernández, Gabriela Molina-Olvera, José Manuel Vega-Riquer, Griselda García-Alonso, Geidy Rodríguez-Version, Francisco Xavier Olmos-García.
