# Supplementary material for: Metals, Nanoparticles, Particulate Matter, and Cognitive Decline
Source: Front Neurol. 2022 Jan 21;12:794071. doi: 10.3389/fneur.2021.794071 (PMC8815025; doi:10.3389/fneur.2021.794071)
Supplement: Supplementary file 1 [file Table_1.docx]

**Supplemental Table 1.** Current Air quality standards for PM_2.5_ for Mexico and the US EPA and the respective WHO Guidelines~~.~~

| **Pollutant** | **Mexico** | **US EPA** | **WHO** |
| --- | --- | --- | --- |
|  | **Max limit (μg/m^3^)** | | **Guidelines (μg/m^3^)** |
| PM_2.5_ | 45 (24-h mean)  12 (annual mean) | 35 (98th percentile, averaged over 3 years)  12 (annual mean, averaged over 3 years) | 25 (24-h mean)  10 (annual mean) |
